# Forward momentum: progressive change through diversity equity and inclusion initiatives in academic health care

**DOI:** 10.1093/jncics/pkaf029

**Published:** 2025-03-14

**Authors:** Chaitanya Kalavagunta, Bansi Savla, Jessica White, Dominic Bulkley, Anna Dunlap, Renee Kwok, Kennecia Shaw, Michael MacFarlane, Sara Dudley, David Alicia, Kimberly Marter, Rivka Leichter, Cameron Chason, Søren M Bentzen, William Regine, Melissa Vyfhuis

**Affiliations:** Department of Radiation Oncology, University of Maryland School of Medicine, Baltimore, MD 21201, United States; Department of Radiation Oncology, University of Maryland School of Medicine, Baltimore, MD 21201, United States; Department of Radiation Oncology, University of Maryland School of Medicine, Baltimore, MD 21201, United States; Department of Radiation Oncology, University of Maryland School of Medicine, Baltimore, MD 21201, United States; Department of Radiation Oncology, University of Maryland School of Medicine, Baltimore, MD 21201, United States; Department of Radiation Oncology, University of Maryland School of Medicine, Baltimore, MD 21201, United States; Department of Radiation Oncology, University of Maryland School of Medicine, Baltimore, MD 21201, United States; Department of Radiation Oncology, University of Maryland School of Medicine, Baltimore, MD 21201, United States; Department of Radiation Oncology, University of Maryland School of Medicine, Baltimore, MD 21201, United States; Department of Radiation Oncology, University of Maryland School of Medicine, Baltimore, MD 21201, United States; Department of Radiation Oncology, University of Maryland School of Medicine, Baltimore, MD 21201, United States; Department of Radiation Oncology, University of Maryland School of Medicine, Baltimore, MD 21201, United States; Department of Radiation Oncology, University of Maryland School of Medicine, Baltimore, MD 21201, United States; Department of Radiation Oncology, University of Maryland School of Medicine, Baltimore, MD 21201, United States; Department of Radiation Oncology, University of Maryland School of Medicine, Baltimore, MD 21201, United States; Department of Radiation Oncology, University of Maryland School of Medicine, Baltimore, MD 21201, United States

## Abstract

**Background:**

This study evaluates the impact of a 2-year Diversity, Equity, and Inclusion (DEI) intervention program within a radiation oncology department. We analyzed employee perceptions of inclusivity, bias, training, and career development, recognizing the challenges of assessing DEI initiatives in an evolving sociopolitical context.

**Methods:**

A voluntary survey was distributed in 2021 and 2023. The survey assessed 4 DEI domains: Inclusivity, Department Bias, Training/Education, and Career Development. Responses were analyzed using nonparametric tests. DEI initiatives included implicit bias training, allyship training, book clubs, anonymous feedback platforms, and a DEI website.

**Results:**

Survey completion rates were 40% (2021) and 50% (2023). Significant improvements were observed in Inclusivity (3.72 vs 3.91, *P* = .042), Training/Education (3.57 vs 4.14, *P* < .001), and Career Development (3.39 vs 3.60, *P* = .019). Department Bias showed no significant change (*P* = .130). Anti-DEI sentiment increased in 2023, highlighting challenges in fostering inclusivity. Subgroup analyses revealed improvements for Black employees but persistent disparities for women.

**Conclusions:**

This exploratory study suggests that targeted DEI initiatives can improve employee perceptions of workplace culture in academic health-care settings. Notably, the program improved perceptions of inclusivity, training opportunities, and career development. However, persistent gender inequities in training and career development highlight the need for monitoring and focused efforts. These findings can inform future DEI strategies and underscore the importance of continued vigilance in promoting an inclusive work environment. Future research should explore the program’s downstream effects on patient care, clinical trial enrollment, and residency enrollment.

## Introduction

Diversity, Equity and Inclusion (DEI) initiatives have emerged as critical strategies for fostering equity and belonging in academic settings, particularly in health care.^1^^,^^2^ Within radiation oncology, these programs aim to address systemic inequities and create an inclusive environment for diverse stakeholders.^3^ Although the potential benefits of DEI initiatives are widely acknowledged, their implementation and outcomes are complex, influenced by cultural, institutional, and societal factors. DEI education has the potential to address inequities, promote fairness, and contribute to the effectiveness of academic institutions.^4^ As health care evolves toward patient-centered care and caters to increasingly diverse populations,^5^ the need for robust DEI initiatives becomes even more critical.^6^ With the US population becoming more racially and ethnically diverse over the past decade,^7^ the demand for culturally competent care has also surged.^8^^,^^9^ Consequently, some medical schools and residency programs are revamping their curriculum to integrate DEI education,^1^ equipping future physicians with the tools necessary to serve culturally diverse patient populations with compassion and equity.^10^^,^^11^

Although DEI initiatives offer potential benefits for health-care institutions and patients, their implementation has faced limitations in certain states. Opposition to these programs is growing, driven by concerns about their necessity and effectiveness in academic and organizational settings.^12^^,^^13^ This resistance reflects a broader societal shift in attitudes toward social justice and inclusivity, potentially hindering progress in these areas.^14^ The decision by some institutions to downplay or eliminate DEI initiatives highlights the significant impact of the political climate on academic medicine, as exemplified by recent events within the American Academy of Dermatology.^15^ Although the Academy ultimately rejected a resolution to dismantle DEI programs and plans to expand these efforts,^16^ similar sentiments have emerged across the country.^17^^,^^18^ These anti-DEI efforts can negatively impact resource allocation, institutional support, and ultimately the overall commitment to fostering inclusive environments.^19^ As political priorities change, DEI programs may compete for resources and attention, potentially leading to their reconsideration or discontinuation.^20^ Therefore, it is crucial to have evidence demonstrating the positive impact of DEI initiatives within health-care departments and organizations to support their continuation in states where they are limited or prohibited.

Although the benefits of DEI initiatives are generally recognized, data are limited on the long-term impact of DEI training or educational events in academic health-care settings.^21^ Therefore, we chose to evaluate the impact of DEI initiatives on workplace culture within an academic radiation oncology department over a 2-year period. Understanding how DEI initiatives are implemented and how they influence faculty and staff’s workplace perceptions is crucial for fostering a truly inclusive academic environment.^22^ This study aims to evaluate the impact of DEI initiatives on workplace culture within an academic radiation oncology department over a 2-year period, addressing the need for evidence-based data to support the continued implementation of such programs amidst growing opposition. It should be noted that this study aims to observe trends in DEI indices over time without seeking to establish causation. By focusing on trends, we acknowledge that causal relationships cannot be inferred from the data collected.

## Methods

### Study design and participants

This study utilised a descriptive, exploratory design to evaluate DEI initiatives implemented within a radiation oncology department. The survey was adapted from the Association of International Certified Professional Accountants Private Companies Practice Section) Diversity and Inclusion Toolkit, Appendix S1,^23^^,^^24^ which was chosen due to its established relevance in assessing workplace inclusion. Voluntary surveys were distributed to all departmental employees in January 2021 and June 2023. Respondents included faculty, administrative staff, radiation therapists, dosimetrists, physicists, and nurses. To ensure anonymity, the surveys did not collect information on specific job roles, and participation was not incentivized. Staff was required to answer all of the survey questions, with optional reporting of participant demographics pertaining to age (18-24, 25-34, 35-44, 45-54, 55+ years old), sex (male vs female), race (White, Black, Latinx/Hispanic, Asian/Pacific Islander, Multiracial), seniority in the department (<1-3, 4-9, 10+ years), and location of employment (Main Academic Center vs Community sites) within the radiation oncology various sites. Employees were given the opportunity to provide open-ended comments on each of the questions and at the end of the questionnaire.

### Development and validation of the DEI index score

#### Item generation and scoring

The survey questions were grouped and analyzed into 4 broad categories, and a DEI Index Score (DIS) was calculated for the following: Inclusivity, Department Bias, DEI Training/Education, and Career Development. The survey questions and their grouping can be found in Appendix S1. The DIS was developed internally based on a comprehensive departmental survey designed to assess various DEI aspects within the department. Each survey question was followed by a 5-point Likert scale ranging from “strongly disagree” to “strongly agree.” This scale allowed for a quantitative assessment of respondents’ attitudes and perceptions.

#### Scoring and index calculation

To calculate the DIS, responses to individual questions were assigned numerical values based on their position on the Likert scale. For example, “strongly disagree” was assigned a value of 1, and “strongly agree” was assigned a value of 5. The scores for questions within each theme were then averaged to create a subindex for that theme. For example, the subindex for Inclusivity would be calculated by averaging the scores for questions 1, 2, 3, and 5. Finally, the subindices for all 4 themes were averaged to create the overall DIS. A higher DIS score indicates a more positive perception of DEI within the department.

#### Validation

The DIS was internally validated through several methods: (1) Content validity: the survey questions were reviewed by DEI committee members to ensure that they adequately covered the relevant dimensions of DEI. (2) Construct validity: The DIS was compared with other measures of DEI to assess its construct validity by the thematic analysis coders. This involved correlating the DIS with existing DEI measures or examining how it relates to relevant outcomes, such as employee satisfaction or retention.

### Data collection and metrics

The survey collected data on perceptions of DEI initiatives, general workplace climate, and experiences with inclusion. Attendance metrics for DEI-related events were not collected, which limits our ability to directly correlate participation in DEI initiatives with survey outcomes. This limitation is explicitly acknowledged and addressed in the “Discussion” section.

### Ethical considerations

This study utilised anonymized survey data and focused on nonclinical topics. As such, it was determined to be exempt from formal Institutional Review Board review. The exemption is based on the nature of the data and the scope of the study, which posed minimal risk to participants.

### Statistical analysis

Nonparametric tests were employed due to the ordinal nature of the data and nonnormal distributions. The use of nonparametric methods is particularly appropriate for ordinal data and small sample sizes, as they do not assume normality and are robust to outliers.^25^ χ^2^ testing was used to assess differences between employee demographics and specific survey questions. The Mann-Whitney U-test was used to calculate differences between grouped indices and specific survey question comparisons by year, as it is suitable for analyzing differences between 2 independent groups when data are not normally distributed.^26^ The Kruskal-Wallis test was used to assess significance between seniority and age categories, as it generalizes the Mann-Whitney *U*-test to more than 2 groups, maintaining validity for ordinal and skewed data distributions.^27^ Statistical significance was determined using a 2-tailed *P* < .05 threshold. Analyses were conducted using SPSS software (SPSS Statistics for Mac, version 29.0.1.0, IBM, Armonk, NY).

### Thematic analysis

A thematic analysis was done utilising employee’s comments provided in the 2021 and 2023 surveys. Four coders met multiple times to discuss the comments given the 2 years, deciding on persistent themes that were repeatedly mentioned in 2021 and other major topics revealed in 2023. Once recurring themes were identified, the 4 coders assessed how the department faired overall (very poor to very good) based on the nature of the employee’s comments for each year using a 5-point scale (see Appendix S2). Kappa testing was done to determine intercoder reliability. Individual employee comments or quotes are not included in this article due to the absence of prior permission obtained during the survey data collection process.

### DEI interventions

The department implemented 5 DEI initiatives over 2.5 years:

1. *Implicit bias training*: A mandatory training for faculty and recommended for staff, focusing on recognizing and mitigating unconscious biases.
2. *Allyship at work training*: This optional yet strongly encouraged training focused on allyship skills, including active listening, empathy, and advocacy for marginalized groups, aiming to foster allyship within the department.
3. *Book club*: An open-to-all initiative, the book club encouraged discussions on DEI topics through selected readings. Although specific attendance data were not tracked, feedback indicated participation across various staff demographics, fostering shared understanding of DEI themes.
4. *Feedback corner*: An anonymous platform was created for staff to provide DEI-related feedback. Submitted comments were reviewed by the DEI committee to address concerns and shape future initiatives.
5. *DEI website*: Accessible via the School of Medicine’s departmental page, the DEI website served as a central hub for resources, event details, and DEI materials, promoting awareness and engagement. Although individual usage data were not monitored, the website functioned as a key resource for supporting an inclusive department culture.

## Results

### Survey participation and representation

Survey response rates were 41% (135/332) in January 2021 and 50% (201/406) in June 2023, respectively. Department demographics are summarized in Table 1. Despite the approximate 20% increase in employees hired over this 2.5-year period, when comparing values between the 2 years, no significant differences were observed in terms of race, gender, age bracket, location, or seniority indicating a relatively stable representation of employees in the department over the 30-month period. A summary of DEI-related initiatives implemented in the department is listed in Table 2. Tables S1-S4 present department survey responses from 2021 and 2023 stratified by race (Table S1), gender (Table S2), seniority or years within the department (Table S3), and location (Table S4). It should be noted that as survey participation was voluntary, these findings may not be fully generalizable to the entire department without representative sampling.

**Table 1.** Department demographics when surveys were given in 2021 and 2023.^a^

	2021 (*n* = 135)	2023 (*n* = 201)	*P* ^b^
**Percent completion**	40.7%	49.5%	
**Race No. (%)**			
White	84 (62.2)	107 (53.2)	.352
Black	18 (13.3)	37 (18.4)	
Asian	14 (10.4)	27 (13.4)	
Latinx/Hispanic	2 (1.5)	3 (1.5)	
Multiracial	1 (0.7)	5 (2.5)	
Missing	16 (11.9)	22 (10.9)	
**Gender**			
Men	31 (23)	61 (30.3)	.233
Women	90 (66.7)	130 (64.7)	
Missing	14 (10.4)	10 (5)	
**Age**			
18-24 years	4 (3)	2 (1)	.468
25-34 years	31 (23)	24 (22.4)	
35-44 years	39 (28.9)	65 (32.3)	
45-54 years	28 (20.7)	52 (25.9)	
55+ years	22 (16.3)	26 (12.9)	
Missing	11 (8.1)	11 (5.5)	
**Time employed in the department**			
<1 year	12 (8.9)	26 (12.9)	.305
1-3 years	29 (21.5)	30 (14.9)	
3-6 years	35 (25.9)	49 (24.4)	
6-10 years	20 (14.8)	42 (20.9)	
10+ years	29 (21.5)	43 (21.4)	
Missing	16 (11.9)	11 (5.5)	
**Location**			
Main campus	86 (63.7)	137 (68.2)	.088
Community sites	49 (36.3)	51 (25.4)	
Missing	0 (0)	13 (6.5)	

a Total number of departmental employees in 2021 and 2023 are 332 and 406, respectively.

b χ^2^ testing was used to assess differences between demographic categories.

**Table 2.** Summary of Diversity, Equity and Inclusion (DEI) training, education, and events offered to the department from January 2021 to December 2023.

**DEI training/Education**
*Unconscious/Implicit bias training* (est. February 2021) Program offered by the School of Medicine to all faculty, made accessible to all employees in the Department by DEI committee efforts *Allyship at work training program* (www.leanin.org; est. January 2023) Program that trains employees how to recognize their privilege and positional power to help cultivate an inclusive work place. DEI committee moderates the sessions twice a year, total 6 h each session
**DEI events**
*DEI speaker series* (est. May 2022) Invited speaker gives a 45-min lecture on DEI topics, followed by a 15-min question and answer session open to all attendees, then meets with residents and faculty afterward for a more informal discussion *DEI book/movie club* (est. November 2021) Quarterly event lasting 1 h, discussing a diverse range of books and movies focusing on the voices of historically marginalized groups
**DEI initiatives**
*DEI feedback corner* (est. September 2021) An application developed so colleagues can anonymously voice any DEI concerns or needs in the department *DEI department website* (est. September 2022) Designed and established a DEI website for the department, stating our mission statement, goals, members, and events *Recruitment/Retention efforts* DEI session/talk incorporated into orientation for new trainees (medical/physics residents, dosimetry students, new hires) Incorporating a holistic approach for medical/physics residency and faculty review/interviews Training/encouraging senior faculty to *mentor* junior faculty, intentionally focusing on underrepresentative groups

### DEI survey results by year

**Figure 1.**
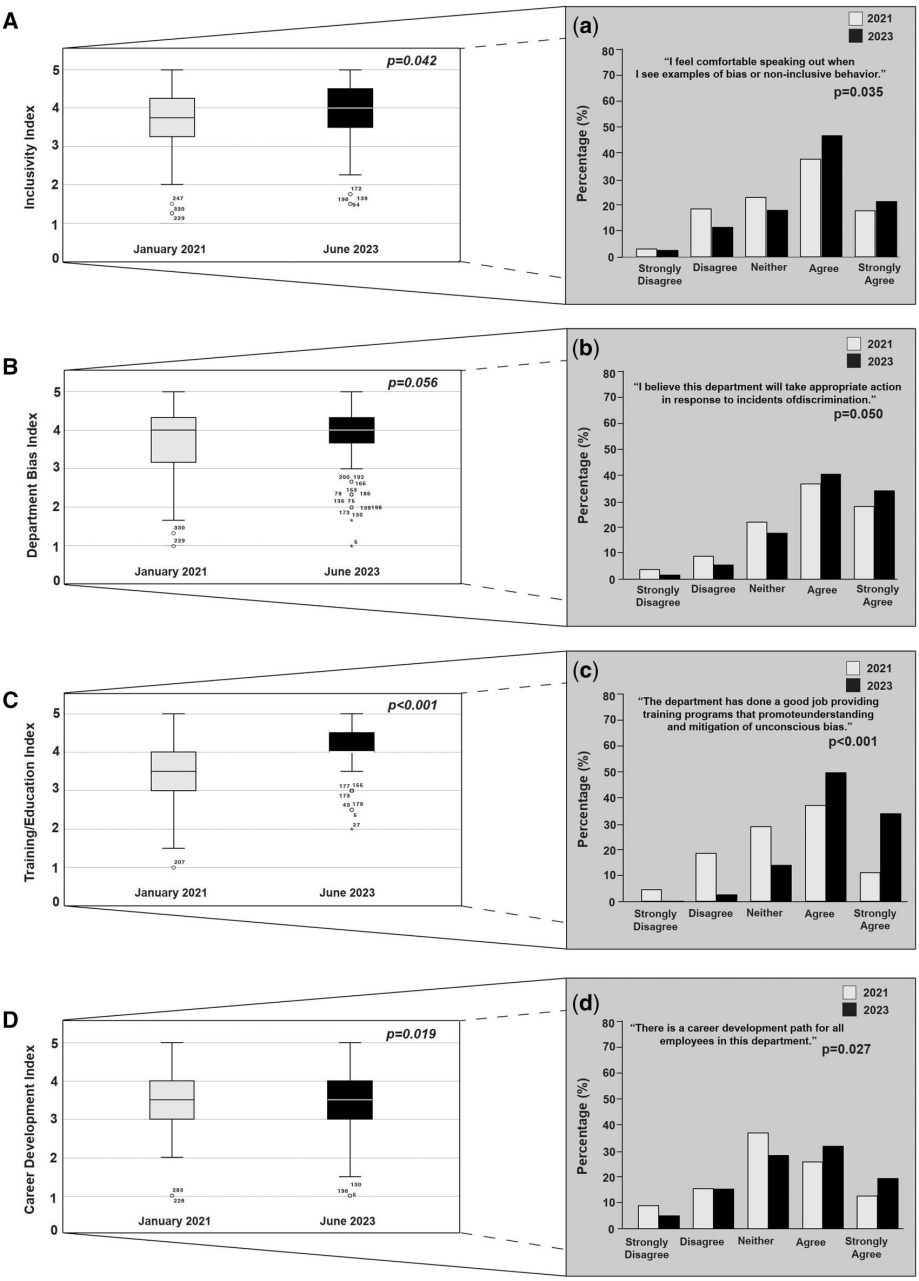
DEI Index Scores (DISs) stratified by when the survey was done (2021 vs 2023). Box and whisker plots show grouped survey questions in classified DEI categories (**A-D**) and bar graphs highlight specific survey questions (**a-d**) from that category that illustrate differences between the 2 years. Each box and whisker plot shows the minimum and maximum using the furthest horizontal lines, the upper and lower quartile of data in the shaded box, and the median as the line within the shaded box. Outliers are represented as individual data points. Exact *P* values are displayed on the plots and insets, with statistically significant differences defined as *P* ≤ .05.

DEI indices for grouped survey categories comparing 2021 and 2023 are summarized in Figure 1. Statistically significant improvements were observed in Inclusivity (mean 2021 score = 3.72 vs mean 2023 score = 3.91; *P* = .042; Figure 1, A), DEI Education/Training (mean 2021 score = 3.57 vs mean 2023 score = 4.14; *P* < .001; Figure 1, C) and Career Development scores (mean 2021 score = 3.39 vs mean 2023 score = 3.60; *P* = .019; Figure 1, D) within the department after DEI-specific interventions and activities were introduced. Specifically, 56% of employees agreed or strongly agreed that they felt comfortable speaking out against noninclusive behavior in early 2021, but this significantly improved to 68% 30 months later (Figure 1, a). The largest improvement in DEI indices was in DEI Training/Education, where 84% of staff agreed or strongly agreed in 2023 that the department provided effective training on unconscious bias, compared with 48% in 2021 (Figure 1, c). Similarly, 39% of employees felt there was a clear path for career development in 2021, which increased to 51% in 2023 (Figure 1, d).

There were no statistically significant changes in the Department Bias Index between 2021 and 2023. In 2023, 65% of employees reported not observing bias or noninclusive behavior, compared with 59% in 2021; however, this difference was not statistically significant (*P* = .130), suggesting that the variation may not represent a meaningful change. Notably, the percentage of employees who strongly agreed or agreed that the department would take appropriate action against discrimination increased from 65% in 2021 to 75% in 2023, with this difference approaching statistical significance (*P* = .050; Figure 1, b).

### DEI survey results by year and demographics

**Figure 2.**
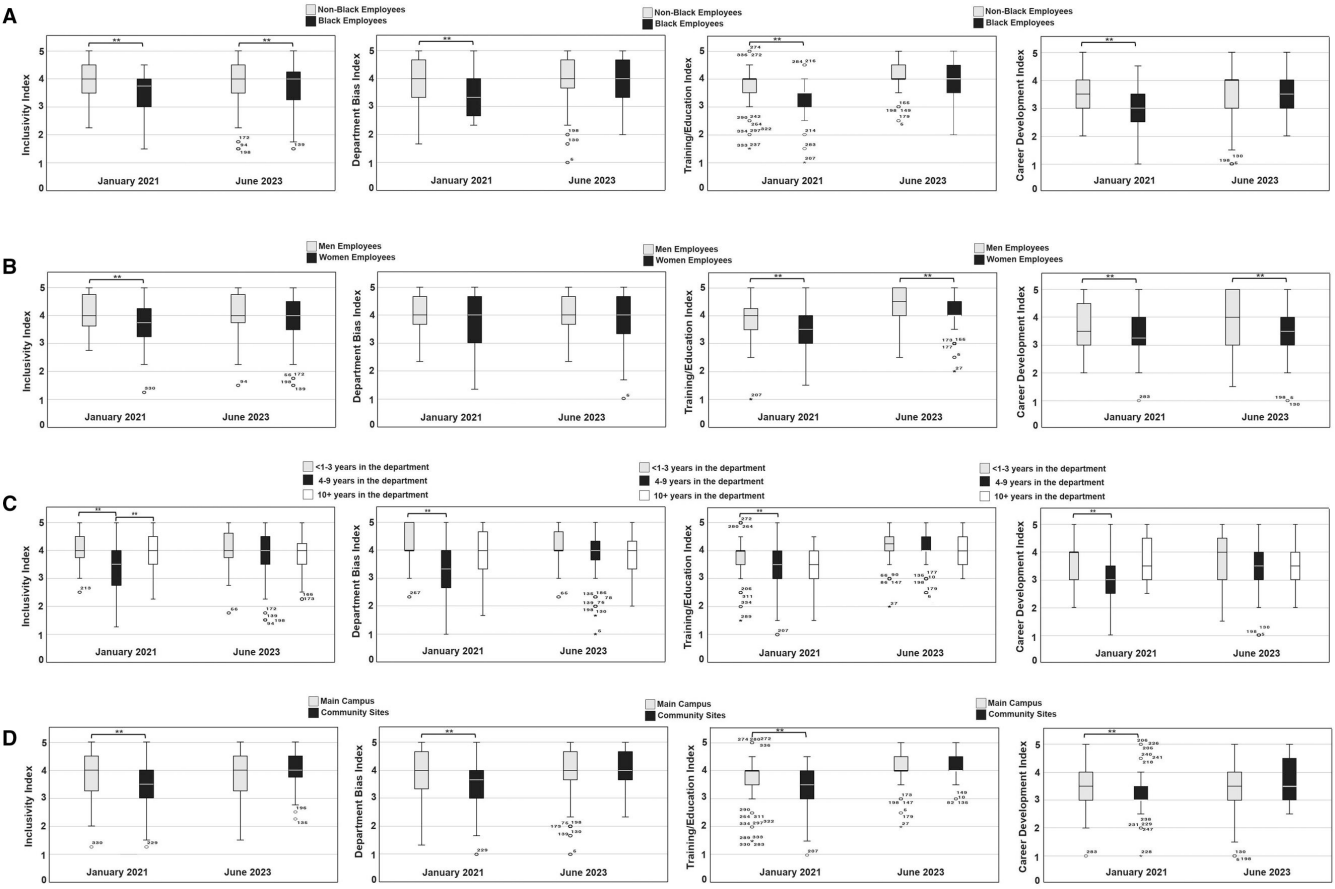
DEI Index Scores (DISs) stratified by when the survey was done (2021 vs 2023) and various demographic categories (**A-D**). **P ≤ .05.

DEI indices for selected demographics and year are summarized in Figure 2. In 2021, compared with White colleagues, Black employees had a significantly lower DEI index scores across all categories. By 2023, these differences were no longer statistically significant in all but 1 category (Inclusivity Index) (Figure 2, A).

In 2021, women had significantly lower DEI indices scores for Inclusivity (Figure 2, B, mean score = 3.75 vs 4.13; *P* = .022), DEI Training/Education (Figure 2, B, mean score = 3.49 vs 3.85; *P* = .030) and Career Development (Figure 2, B, mean score = 3.17 vs 3.77; *P* = .021) compared with men. By 2023, these differences were no longer statistically significant for Inclusivity, though gaps remained in Training/Education (Figure 2, B, mean score = 4.08 vs 4.31; *P* = .015) and Career Development (Figure 2, B, mean score = 3.51 vs 3.86; *P* = .018). In 2021, employees with 4-9 years of tenure reported significantly lower DEI scores compared with their peers in other tenure groups. This difference was no longer significant by 2023 (Figure 2, C).

### DEI survey thematic analysis on employee comments

**Figure 3.**
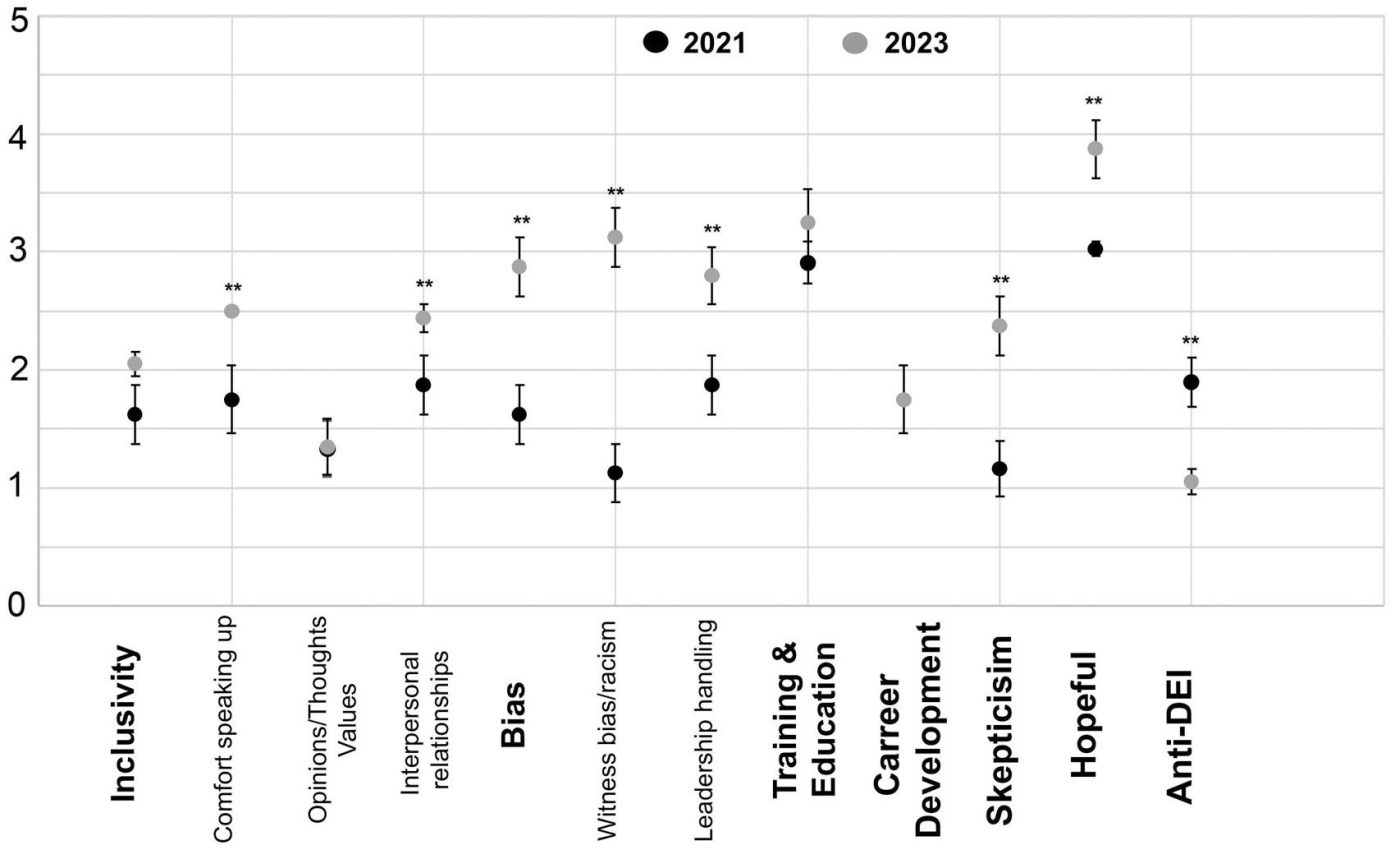
Average coder scores of how the department performed (1 = very poor to 5 = very good) on persistent themes uncovered during the thematic analysis based on employee comments found on the survey in 2021 (**black circles**) and 2023 (**gray circles**). Error bars represent the SD of the coder scores.

Employees were encouraged to provide written feedback on the 2021 and 2023 surveys. Using a thematic analysis approach, 4 coders discovered persistent themes that were commented on during each survey session (Figure 3). Intercoder reliability ranged from fair to near-perfect agreement (Table 3). Most metrics improved between 2021 and 2023, including employees’ comfort in speaking up about DEI issues (mean score 2021 = 1.75; mean score 2023 = 2.50; *P* = .029) and a decrease in witnessing bias or racism (mean score 2021 = 1.13; mean score 2023 = 3.13; *P* = .029; Figure 3). Employees also reported improved leadership handling of DEI-related circumstances, increasing from a mean score of 1.88 in 2021 to 2.80 in 2023. Skepticism about true change in the department decreased (mean score 2021 = 1.16; mean score 2023 = 2.38), whereas hopefulness about meaningful change increased (mean score 2021 = 3.03; mean score 2023 = 3.88; *P* = .029 Figure 3). Notably, there was a rise in anti-DEI sentiments, with more comments advocating for limiting or abolishing DEI training in 2023 (mean score 2021 = 1.90; mean score 2023 = 1.06; *P* = .029).

**Table 3.** Kappa values between coders for each survey year.^a^^,^^b^

	Kappa value	*P*
**Year: 2021**		
Coder 1-Coder 2	0.684	<.001
Coder 1-Coder 3	0.341	.030
Coder 1-Coder 4	0.769	<.001
Coder 2-Coder 3	0.407	<.013
Coder 2-Coder 4	0.605	.002
Coder 3-Coder 4	0.404	.014
**Year: 2023**		
Coder 1-Coder 2	0.822	<.001
Coder 1-Coder 3	0.676	<.001
Coder 1-Coder 4	0.773	<.001
Coder 2-Coder 3	0.717	<.001
Coder 2-Coder 4	0.955	<.001
Coder 3-Coder 4	0.736	<.001

a Four coders (labeled 1-4) scored, using a 5-point scale, how the department performed, from "very poor" to "very good," on each theme identified in the thematic analysis (Appendix S1).

b Cohen’s weighted Kappa was done on each pair of coders.

## Discussion

This study investigated the impact of a multipronged 2-year DEI initiative within an academic radiation oncology department. By analyzing survey data and employee comments, we sought to understand how these initiatives influenced employees’ sense of inclusion, perceived bias, access to training opportunities, and career development pathways.

### Key findings and implications

Our findings offer valuable insights into the effectiveness of the implemented interventions and highlight areas for continued focus in fostering a more inclusive departmental culture. A statistically significant rise in the Inclusivity score suggests that employees were able to behave in a more authentic manner with their colleagues and felt that their professional opinions were more valued after DEI events were implemented. The thematic analysis corroborated this trend (Figure 3), revealing a greater sense of comfort among employees in speaking up about DEI concerns. This aligns with prior research by Downey et al,^28^ which found that targeted diversity training programs fostered a more inclusive work environment in a health-care setting. However, in comparison, our work is more nuanced, delving into important demographic differences that can truly help us tailor and design future DEI initiatives.

The most striking improvement was observed in the DEI Training/Education score. This suggests that the implemented training programs were successful in raising awareness and fostering understanding of unconscious bias and other DEI-related topics. This finding aligns with research by Burnes et al^29^ which demonstrated the effectiveness of educational interventions in mitigating discriminatory behavior within the workplace. By equipping employees with the knowledge and tools to recognize and address bias, the DEI initiatives likely contributed to a more equitable work environment. A particularly encouraging finding was the diminishing disparity in DEI indices between Black employees and their White colleagues (Figure 2). Similarly, the gender gap in some indices narrowed over time. This suggests that the implemented DEI initiatives were successful in addressing the specific needs of underrepresented groups within the department.

Despite the overall positive trends, the study reveals persistent inequities between men and women. Although women’s DEI scores improved across the board, they still lagged behind men’s scores in Training/Education and Career Development. This finding underscores the need for further targeted efforts to ensure equity in these areas. Our thematic analysis highlights women’s concerns about professional development opportunities suggesting that a one-size-fits-all approach might not be sufficient. The department could explore tailored training programs or mentorship opportunities specifically designed to address the unique challenges and aspirations of women within the department, especially Black women.^30^ Similarly, the initial inequities in DEI scores based on seniority highlight the importance of ensuring inclusivity across all career stages. For example, early career employees might benefit from onboarding programs that emphasize inclusion, whereas midcareer employees might be more interested in leadership development opportunities that consider diversity and equity. Although these initial differences disappeared by 2023, it suggests that the original rollout of DEI initiatives may not have resonated as effectively with employees that had 4-9 years of experience. The successful elimination of the initial disparity in DEI perceptions between the main academic campus and community sites is also a notable achievement. This demonstrates that the implemented DEI initiatives were effective in fostering a more cohesive departmental culture across all locations. By creating a sense of shared purpose and belonging, regardless of physical location, the department is promoting a more collaborative and supportive work network.

The overall Departmental Bias score remained statistically similar between the years (Figure 1). Although fewer employees observed instances of bias in 2023, this might indicate that bias is still present but less overt or frequently observed. One possibility is that the specific DEI activities implemented may not have directly addressed the root causes of bias within the department. Another possibility is that a longer time frame might be necessary to observe more substantial changes in employee perceptions and behaviors.

One unexpected result was the emergence of anti-DEI sentiment in employee comments from 2023. Although the reasons for this remain unclear, it underscores the importance of ongoing communication and education about the goals and benefits of DEI initiatives. This finding aligns with broader societal trends, where DEI initiatives can sometimes provoke backlash in certain cultural contexts. Perhaps a qualitative study involving focus groups or interviews could shed light on the specific concerns or misunderstandings driving this sentiment. This would allow employees to voice their experiences with bias and their perspectives on the DEI initiatives in a more nuanced way. By gaining a clearer understanding of the nature of the bias and the source of the anti-DEI sentiment, the department can develop more targeted strategies to address these challenges.

It is important to acknowledge that the challenges of DEI in oncology may differ from other health-care settings. For example, addressing patient diversity, ensuring equitable access to care, and navigating the unique challenges of oncology recruitment require specific considerations. Future surveys should be modified to address these oncology-specific issues. Future studies should employ more rigorous methodologies, such as experimental or longitudinal designs, to better assess causality. This could involve pre- and postintervention matched cohorts or, where feasible, randomized controlled trials to determine the specific impact of individual DEI interventions.

### Limitations

This study has several limitations that must be considered when interpreting its findings. One significant limitation is the lack of attendance tracking for DEI interventions, which restricts the ability to directly link participation in specific initiatives with changes in employee perceptions. Future research should incorporate robust participation metrics to strengthen the connection between intervention engagement and observed outcomes.

Although the DEI Index Score underwent thorough internal validation through expert review and thematic analysis, it lacks external validation, which limits its broader applicability. Validating the index against established DEI metrics in future studies will enhance its reliability and generalizability. Similarly, the reliance on self-reported survey data introduces the potential for bias, as the sample may overrepresent employees who are supportive of DEI initiatives while underrepresenting skeptics. To address this, future studies should consider stratified sampling techniques or incentivized participation to improve response rates and reduce potential biases.

Additionally, this study did not explicitly explore the experiences of sexual or gender minorities within the department. These groups may face unique challenges and barriers to inclusion that were not captured in the survey. Future research should include targeted questions to address the specific needs and concerns of LGBTQ+ employees, such as their experiences with discrimination, access to resources, and perceptions of inclusivity.

The study’s scope also limits the generalizability of its findings. Conducted within a single academic radiation oncology department, the results may be influenced by factors unique to this environment, including the department’s preexisting culture, leadership support for DEI, and workforce composition. As Paradis et al^31^ noted, the structure, resources, and recognition of DEI work vary significantly across institutions, potentially affecting the effectiveness of DEI initiatives. Expanding research to include multiple institutions and diverse settings will help assess the transferability of these findings to broader health-care environments.

Other methodological limitations include optional survey comment sections, varying levels of engagement across the 2 survey periods, and relatively low response rates, which may limit the representativeness of the findings. Furthermore, although thematic analysis provided valuable insights, it relied on coded themes, potentially overlooking nuances in employee experiences. Larger sample sizes and more diverse health-care settings in future studies would strengthen the validity and applicability of these findings. Longitudinal studies could also track the sustainability of positive changes over time, whereas qualitative approaches, such as in-depth interviews or focus groups, could provide a richer understanding of employee perceptions of DEI initiatives.

Secular trends and broader cultural shifts in DEI awareness and activism may have influenced the observed improvements, making it challenging to isolate the effects of departmental interventions. Exploring how external societal changes intersect with internal initiatives will provide a clearer understanding of the factors driving change.

Finally, it is crucial to acknowledge that this study is exploratory and descriptive in nature. The findings offer valuable insights into the effectiveness of the implemented interventions and highlight areas for continued focus in fostering a more inclusive departmental culture. However, causal links between specific interventions and observed outcomes cannot be definitively established based on this study design.

### Future research

To build on the findings of this study, future research should explore the impact of DEI initiatives on measurable outcomes such as patient satisfaction, health-care equity, and organizational performance. Establishing clear links between DEI efforts and these metrics will strengthen the case for the value of such initiatives in health care. Additionally, investigating the intersectionality of identity factors, such as race, gender, and seniority, in shaping DEI experiences will provide a more comprehensive understanding of inequities within academic health-care settings. By examining how multiple dimensions of identity intersect and interact, researchers can develop targeted interventions to address the specific challenges faced by marginalized groups. These efforts will not only enhance DEI initiatives but also contribute to fostering inclusive environments that benefit employees, patients, and the broader health-care system.

### Recommendations

Based on the current results of our study, several recommendations can be made to guide future DEI initiatives and organizational practices in general academic health-care settings: *Continued evaluation*: Conduct periodic assessments of DEI initiatives through surveys, focus groups, and feedback mechanisms to monitor progress, identify areas for improvement, and maintain accountability. Share findings transparently with all employees. *Intersectional approach*: Address the unique challenges faced by individuals at the intersections of multiple identity factors, such as race, gender, and seniority, by tailoring interventions to meet diverse workforce needs. *Leadership commitment*: Foster visible and sustained leadership support for DEI goals by allocating resources and holding leaders accountable for achieving diversity and equity targets. *Training and development*: Develop tailored training programs, including unconscious bias training, allyship at work or similar training programs,^32^ and mentorship opportunities, to address employee-specific concerns, promote career advancement, and create a supportive work environment.

## Conclusions

This study provides evidence that well-designed and implemented DEI initiatives can contribute to a more inclusive and equitable work environment in academic health-care settings. Our findings add to the existing body of research highlighting the benefits of such initiatives for employee perceptions, overall workplace culture, and the professional development of a diverse workforce. However, ongoing efforts are necessary to address persisting disparities, counteract resistance to DEI initiatives, and ensure the long-term sustainability of positive changes. Further research is crucial to expand the generalizability of these findings and to gain deeper insights into employee experiences with DEI initiatives within academic health-care settings.

## Supplementary Material

pkaf029_Supplementary_Data

## Data Availability

Research data will be shared upon request to the corresponding author.
