# Supplementary material for: Forward momentum: progressive change through diversity equity and inclusion initiatives in academic health care
Source: JNCI Cancer Spectr. 2025 Mar 14;9(2):pkaf029. doi: 10.1093/jncics/pkaf029 (PMC11976714; doi:10.1093/jncics/pkaf029)
Supplement: pkaf029_Supplementary_Data [file pkaf029_supplementary_data.docx]

| **Supplemental Table 1.** Department answers when surveys were given in 2021 and 2023 stratified by race. | | | | | | |
| --- | --- | --- | --- | --- | --- | --- |
| ***2021 (N=135) ^a,b^*** | | | | | | |
| **Race n (%)** | **White** | **Black** | **Asian** | **Latino/Hispanic** | **Multiracial** | **p-value** *^c^* |
| **I can be my authentic self at work.** | | | | | | |
| **Strongly Disagree** | 0 (0) | 1 (5.6) | 0 (0) | 0 (0) | 0 (0) | 0.184 |
| **Disagree** | 3 (3.6) | 1 (5.6) | 0 (0) | 0 (0) | 0 (0) |  |
| **Neither** | 8 (9.5) | 5 (27.8) | 2 (14.3) | 1 (50) | 1 (50) |  |
| **Agree** | 47 (56) | 8 (44.4) | 7 (50) | 0 (0) | 0 (0) |  |
| **Strongly Agree** | 26 (31) | 3 (16.7) | 5 (35.7) | 1 (50) | 1 (50) |  |
| **My professional opinions and contributions are considered equally, regardless of my individual characteristics.** | | | | | | |
| **Strongly Disagree** | 0 (0) | 1 (5.6) | 0 (0) | 0 (0) | 0 (0) | 0.060 |
| **Disagree** | 9 (10.7) | 0 (0) | 0 (0) | 0 (0) | 0 (0) |  |
| **Neither** | 9 (10.7) | 4 (22.2) | 1 (7.1) | 1 (50) | 0 (0) |  |
| **Agree** | 46 (54.8) | 13 (72.2) | 6 (42.9) | 0 (0) | 1 (100) |  |
| **Strongly Agree** | 20 (23.8) | 0 (0) | 7 (50) | 1 (50) | 0 (0) |  |
| **I feel comfortable speaking out when I see examples of bias or non-inclusive behavior.** | | | | | | |
| **Strongly Disagree** | 0 (0) | 1 (5.6) | 0 (0) | 0 (0) | 0 (0) | 0.385 |
| **Disagree** | 14 (16.7) | 3 (16.7) | 1 (7.1) | 1 (50) | 0 (0) |  |
| **Neither** | 13 (15.5) | 5 (27.8) | 5 (35.7) | 0 (0) | 1 (100) |  |
| **Agree** | 38 (45.2) | 7 (38.9) | 5 (35.7) | 1 (50) | 0 (0) |  |
| **Strongly Agree** | 19 (22.6) | 2 (11.1) | 3 (21.4) | 0 (0 | 0 (0) |  |
| **I observe examples of bias or non-inclusive behavior on a regular basis in this department.** | | | | | | |
| **Strongly Disagree** | 21 (25) | 1 (5.6) | 5 (35.7) | 1 (50) | 0 (0) | 0.140 |
| **Disagree** | 37 (44) | 7 (38.9) | 4 (28.6) | 0 (0) | 0 (0) |  |
| **Neither** | 12 (14.3) | 2 (11.1) | 4 (28.6) | 0 (0) | 0 (0) |  |
| **Agree** | 10 (11.9) | 7 (38.9) | 1 (7.1) | 1 (50) | 1 (50) |  |
| **Strongly Agree** | 4 (4.8) | 1 (5.6) | 0 (0) | 0 (0) | 0 (0) |  |
| **The department provides an environment for the free and open expression of ideas, opinions and beliefs.** | | | | | | |
| **Strongly Disagree** | 3 (3.6) | 0 (0) | 0 (0) | 1 (50) | 0 (0) | **0.006** |
| **Disagree** | 6 (7.1) | 3 (16.7) | 0 (0) | 0 (0) | 0 (0) |  |
| **Neither** | 16 (19) | 7 (38.9) | 3 (21.4) | 0 (0) | 0 (0) |  |
| **Agree** | 42 (50) | 8 (44.4) | 4 (28.6) | 0 (0) | 1 (100) |  |
| **Strongly Agree** | 17 (20.2) | 0 (0) | 7 (50) | 1 (50) | 0 (0) |  |
| **I believe this department will take appropriate action in response to incidents of discrimination.** | | | | | | |
| **Strongly Disagree** | 0 (0) | 2 (11.1) | 0 (0) | 1 (50) | 0 (0) | **<0.001** |
| **Disagree** | 6 (7.1) | 1 (5.6) | 1 (7.1) | 0 (0) | 1 (100) |  |
| **Neither** | 16 (19) | 7 (38.9) | 2 (14.3) | 0 (0) | 0 (0) |  |
| **Agree** | 35 (41.7) | 6 (33.3) | 4 (28.6) | 0 (0) | 0 (0) |  |
| **Strongly Agree** | 27 (32.1) | 2 (11.1) | 7 (50) | 1 (50) | 0 (0) |  |
| **My supervisor supports diversity by showing commitment to handling matters responsibly and appropriately.** | | | | | | |
| **Strongly Disagree** | 0 (0) | 0 (0) | 0 (0) | 0 (0) | 0 (0) | 0.100 |
| **Disagree** | 7 (8.3) | 0 (0) | 1 (7.1) | 1 (50) | 0 (0) |  |
| **Neither** | 14 (16.7) | 3 (16.7) | 0 (0) | 0 (0) | 1 (100) |  |
| **Agree** | 35 (41.7) | 10 (55.6) | 5 (35.7) | 0 (0) | 0 (0) |  |
| **Strongly Agree** | 28 (33.3) | 5 (27.8) | 8 (57.1) | 1 (50) | 0 (0) |  |
| **The department has done a good job providing training programs that promote understanding and mitigation of unconscious bias.** | | | | | | |
| **Strongly Disagree** | 2 (2.4) | 2 (11.1) | 0 (0) | 0 (0) | 0 (0) | **0.014** |
| **Disagree** | 14 (16.7) | 6 (33.3) | 2 (14.3) | 1 (50) | 0 (0) |  |
| **Neither** | 25 (29.8) | 6 (33.3) | 2 (14.3) | 0 (0) | 0 (0) |  |
| **Agree** | 37 (44) | 3 (16.7) | 4 (28.6) | 0 (0) | 1 (100) |  |
| **Strongly Agree** | 6 (7.1) | 1 (5.6) | 6 (42.9) | 1 (50) | 0 (0) |  |
| **Employees of different backgrounds are encouraged to apply for higher positions.** | | | | | | |
| **Strongly Disagree** | 2 (2.4) | 1 (5.6) | 0 (0) | 0 (0) | 0 (0) | 0.727 |
| **Disagree** | 4 (4.8) | 2 (11.1) | 1 (7.1) | 0 (0) | 0 (0) |  |
| **Neither** | 29 (34.5) | 8 (44.4) | 5 (35.7) | 1 (50) | 0 (0) |  |
| **Agree** | 32 (38.1) | 5 (27.8) | 2 (14.3) | 0 (0) | 1 (100) |  |
| **Strongly Agree** | 17 (20.2) | 2 (11.1) | 6 (42.9) | 1 (50) | 0 (0) |  |
| **There is a career development path for all employees in this department.** | | | | | | |
| **Strongly Disagree** | 5 (6.0) | 2 (11.1) | 1 (7.1) | 0 (0) | 0 (0) | 0.365 |
| **Disagree** | 14 (16.7) | 4 (22.2) | 1 (7.1) | 0 (0) | 0 (0) |  |
| **Neither** | 31 (36.9) | 9 (50) | 4 (28.6) | 1 (50) | 0 (0) |  |
| **Agree** | 24 (28.6) | 3 (16.7) | 3 (21.4) | 0 (0) | 1 (100) |  |
| **Strongly Agree** | 10 (11.9) | 0 (0) | 5 (35.7) | 1 (50) | 0 (0) |  |
| **I have received implicit bias or cultural sensitivity training before.** | | | | | | |
| **Strongly Disagree** | 3 (3.6) | 1 (5.6) | 0 (0) | 0 (0) | 0 (0) | 0.630 |
| **Disagree** | 7 (8.3) | 2 (11.1) | 2 (14.3) | 0 (0) | 0 (0) |  |
| **Neither** | 7 (8.3) | 1 (5.6) | 0 (0) | 0 (0) | 0 (0) |  |
| **Agree** | 53 (63.1) | 12 (66.7) | 5 (35.7) | 1 (50) | 0 (0) |  |
| **Strongly Agree** | 14 (16.7) | 2 (11.1) | 7 (50) | 1 (50) | 0 (0) |  |
| ***2023 (N=201)*** | | | | | | |
| **Race n (%)** | **White** | **Black** | **Asian** | **Latino/Hispanic** | **Multiracial** | **p-value** |
| **I can be my authentic self at work.** | | | | | | |
| **Strongly Disagree** | 2 (1.9) | 2 (5.4) | 1 (3.7) | 0 (0) | 0 (0) | 0.117 |
| **Disagree** | 2 (1.9) | 1 (2.7) | 1 (3.7) | 1 (33.3) | 0 (0) |  |
| **Neither** | 8 (7.5) | 4 (10.8) | 0 (0) | 0 (0) | 1 (20) |  |
| **Agree** | 55 (51.4) | 22 (59.5) | 15 (55.6) | 0 (0) | 1 (20) |  |
| **Strongly Agree** | 40 (37.4) | 8 (21.6) | 10 (37) | 2 (66.7) | 3 (60) |  |
| **My professional opinions and contributions are considered equally, regardless of my individual characteristics.** | | | | | | |
| **Strongly Disagree** | 2 (1.9) | 2 (5.4) | 2 (7.4) | 0 (0) | 0 (0) | 0.228 |
| **Disagree** | 8 (7.5) | 1 (2.7) | 1 (3.7) | 0 (0) | 0 (0) |  |
| **Neither** | 10 (9.3) | 7 (18.9) | 0 (0) | 1 (33.3) | 2 (40) |  |
| **Agree** | 49 (45.8) | 20 (54.1) | 12 (44.4) | 1 (33.3) | 1 (20) |  |
| **Strongly Agree** | 38 (35.5) | 7 (18.9) | 12 (44.4) | 1 (33.3) | 2 (40) |  |
| **I feel comfortable speaking out when I see examples of bias or non-inclusive behavior.** | | | | | | |
| **Strongly Disagree** | 2 (1.9) | 1 (2.7) | 2 (7.4) | 0 (0) | 0 (0) | 0.274 |
| **Disagree** | 12 (11.2) | 5 (13.5) | 0 (0) | 0 (0) | 0 (0) |  |
| **Neither** | 23 (21.5) | 8 (21.6) | 2 (7.4) | 1 (33.3) | 0 (0) |  |
| **Agree** | 47 (43.9) | 17 (45.9) | 13 (48.1) | 0 (0) | 4 (80) |  |
| **Strongly Agree** | 23 (21.5) | 6 (16.2) | 10 (37) | 2 (66.7) | 1 (20) |  |
| **I observe examples of bias or non-inclusive behavior on a regular basis in this department.** | | | | | | |
| **Strongly Disagree** | 32 (29.9) | 7 (18.9) | 8 (29.6) | 1 (33.3) | 0 (0) | 0.253 |
| **Disagree** | 48 (44.9) | 11 (29.7) | 9 (33.3) | 1 (33.3) | 3 (60) |  |
| **Neither** | 13 (12.1) | 14 (37.8) | 4 (14.8) | 1 (33.3) | 1 (20) |  |
| **Agree** | 10 (9.3) | 3 (8.1) | 3 (11.1) | 0 (0) | 1 (20) |  |
| **Strongly Agree** | 4 (3.7) | 2 (5.4) | 3 (11.1) | 0 (0) | 0 (0) |  |
| **The department provides an environment for the free and open expression of ideas, opinions and beliefs.** | | | | | | |
| **Strongly Disagree** | 1 (0.9) | 1 (2.7) | 0 (0) | 0 (0) | 0 (0) | 0.904 |
| **Disagree** | 6 (5.6) | 3 (8.1) | 1 (3.7) | 0 (0) | 0 (0) |  |
| **Neither** | 21 (19.6) | 7 (18.9) | 3 (11.1) | 0 (0) | 1 (20) |  |
| **Agree** | 50 (46.7) | 21 (56.8) | 15 (55.6) | 2 (66.7) | 4 (80) |  |
| **Strongly Agree** | 29 (27.1) | 5 (13.5) | 8 (29.6) | 1 (33.3) | 0 (0) |  |
| **I believe this department will take appropriate action in response to incidents of discrimination.** | | | | | | |
| **Strongly Disagree** | 1 (0.9) | 0 (0) | 1 (3.7) | 0 (0) | 0 (0) | 0.402 |
| **Disagree** | 3 (2.8) | 2 (5.4) | 2 (7.4) | 0 (0) | 1 (20) |  |
| **Neither** | 19 (17.8) | 9 (24.3) | 2 (7.4) | 0 (0) | 0 (0) |  |
| **Agree** | 49 (45.8) | 11 (29.7) | 8 (29.6) | 1 (33.3) | 3 (60) |  |
| **Strongly Agree** | 35 (32.7) | 15 (40.5) | 14 (51.9) | 2 (66.7) | 1 (20) |  |
| **My supervisor supports diversity by showing commitment to handling matters responsibly and appropriately.** | | | | | | |
| **Strongly Disagree** | 1 (0.9) | 1 (2.7) | 0 (0) | 0 (0) | 0 (0) | 0.372 |
| **Disagree** | 5 (4.7) | 1 (2.7) | 0 (0) | 0 (0) | 0 (0) |  |
| **Neither** | 13 (12.1) | 9 (24.3) | 1 (3.7) | 1 (33.3) | 0 (0) |  |
| **Agree** | 48 (44.9) | 7 (18.9) | 13 (48.1) | 1 (33.3) | 3 (60) |  |
| **Strongly Agree** | 40 (37.4) | 19 (51.4) | 13 (48.1) | 1 (33.3) | 2 (40) |  |
| **The department has done a good job providing training programs that promote understanding and mitigation of unconscious bias.** | | | | | | |
| **Strongly Disagree** | 0 (0) | 0 (0) | 0 (0) | 0 (0) | 0 (0) | 0.317 |
| **Disagree** | 2 (1.9) | 2 (5.4) | 0 (0) | 0 (0) | 0 (0) |  |
| **Neither** | 15 (14) | 4 (10.8) | 4 (14.8) | 0 (0) | 0 (0) |  |
| **Agree** | 57 (53.3) | 19 (51.4) | 7 (25.9) | 2 (66.7) | 4 (80) |  |
| **Strongly Agree** | 33 (30.8) | 12 (32.4) | 16 (59.3) | 1 (33.3) | 1 (20) |  |
| **Employees of different backgrounds are encouraged to apply for higher positions.** | | | | | | |
| **Strongly Disagree** | 3 (2.8) | 1 (2.7) | 2 (7.4) | 0 (0) | 0 (0) | 0.340 |
| **Disagree** | 4 (3.7) | 5 (13.5) | 1 (3.7) | 0 (0) | 0 (0) |  |
| **Neither** | 30 (28) | 12 (32.4) | 6 (22.2) | 0 (0) | 0 (0) |  |
| **Agree** | 39 (36.4) | 11 (29.7) | 10 (37) | 3 (100) | 4 (80) |  |
| **Strongly Agree** | 31 (29) | 8 (21.6) | 8 (29.6) | 0 (0) | 1 (20) |  |
| **There is a career development path for all employees in this department.** | | | | | | |
| **Strongly Disagree** | 6 (5.6) | 1 (2.7) | 2 (7.4) | 0 (0) | 0 (0) | 0.487 |
| **Disagree** | 15 (14) | 10 (27) | 1 (3.7) | 0 (0) | 1 (20) |  |
| **Neither** | 31 (29) | 10 (27) | 6 (22.2) | 1 (33.3) | 1 (20) |  |
| **Agree** | 36 (33.6) | 11 (29.7) | 7 (25.9) | 1 (33.3) | 2 (40) |  |
| **Strongly Agree** | 19 (17.8) | 5 (13.5) | 11 (40.7) | 1 (33.3) | 1 (20) |  |
| **I have received implicit bias or cultural sensitivity training before.** | | | | | | |
| **Strongly Disagree** | 0 (0) | 1 (2.7) | 0 (0) | 0 (0) | 0 (0) | 0.639 |
| **Disagree** | 3 (2.8) | 4 (10.8) | 1 (3.7) | 0 (0) | 0 (0) |  |
| **Neither** | 8 (7.5) | 2 (5.4) | 4 (14.8) | 0 (0) | 1 (20) |  |
| **Agree** | 56 (52.3) | 20 (54.1) | 12 (44.4) | 1 (33.3) | 2 (40) |  |
| **Strongly Agree** | 40 (37.4) | 10 (27) | 10 (37) | 2 (66.7) | 2 (40) |  |
| *^a^* Total number of departmental employees in 2020 and 2023 are 332 and 406, respectively. *^b^* Total number of missing race responses was 16 (11.9%) in 2021 and 22 (10.9%) in 2023.  *^c^* Chi-square testing was used to assess differences between demographic categories. | | | | | | |

| **Supplemental Table 2.** Department answers when surveys were given in 2021 and 2023 stratified by gender. | | | |
| --- | --- | --- | --- |
| ***2021 (N=135)* *^a,b^*** | | | |
| **n (%)** | **Men** | **Women** | **p-value** *^c^* |
| **I can be my authentic self at work.** | | | |
| **Strongly Disagree** | 0 (0) | 0 (0) | 0.878 |
| **Disagree** | 1 (3.2) | 3 (3.3) |  |
| **Neither** | 4 (12.9) | 14 (15.6) |  |
| **Agree** | 15 (48.4) | 48 (53.3) |  |
| **Strongly Agree** | 11 (35.5) | 25 (27.8) |  |
| **My professional opinions and contributions are considered equally, regardless of my individual characteristics.** | | | |
| **Strongly Disagree** | 0 (0) | 1 (1.1) | **0.014** |
| **Disagree** | 0 (0) | 10 (11.1) |  |
| **Neither** | 3 (9.7) | 11 (12.2) |  |
| **Agree** | 14 (45.2) | 53 (58.9) |  |
| **Strongly Agree** | 14 (45.2) | 15 (16.7) |  |
| **I feel comfortable speaking out when I see examples of bias or non-inclusive behavior.** | | | |
| **Strongly Disagree** | 0 (0) | 1 (1.1) | 0.255 |
| **Disagree** | 2 (6.5) | 18 (20) |  |
| **Neither** | 8 (25.8) | 17 (18.9) |  |
| **Agree** | 12 (38.7) | 39 (43.3) |  |
| **Strongly Agree** | 9 (29) | 15 (16.7) |  |
| **I observe examples of bias or non-inclusive behavior on a regular basis in this department.** | | | |
| **Strongly Disagree** | 7 (22.6) | 21 (23.3) | 0.206 |
| **Disagree** | 13 (41.9) | 35 (38.9) |  |
| **Neither** | 8 (25.8) | 11 (12.2) |  |
| **Agree** | 3 (9.7) | 18 (20) |  |
| **Strongly Agree** | 0 (0) | 5 (5.6) |  |
| **The department provides an environment for the free and open expression of ideas, opinions and beliefs.** | | | |
| **Strongly Disagree** | 0 (0) | 5 (5.6) | 0.210 |
| **Disagree** | 1 (3.2) | 8 (8.9) |  |
| **Neither** | 5 (16.1) | 20 (22.2) |  |
| **Agree** | 15 (48.4) | 42 (46.7) |  |
| **Strongly Agree** | 10 (32.3) | 15 (16.7) |  |
| **I believe this department will take appropriate action in response to incidents of discrimination.** | | | |
| **Strongly Disagree** | 0 (0) | 3 (3.3) | 0.486 |
| **Disagree** | 1 (3.2) | 8 (8.9) |  |
| **Neither** | 5 (16.1) | 20 (22.2) |  |
| **Agree** | 13 (41.9) | 34 (37.8) |  |
| **Strongly Agree** | 12 (38.7) | 25 (27.8) |  |
| **My supervisor supports diversity by showing commitment to handling matters responsibly and appropriately.** | | | |
| **Strongly Disagree** | 0 (0) | 0 (0) | 0.240 |
| **Disagree** | 0 (0) | 11 (12.2) |  |
| **Neither** | 5 (16.1) | 13 (14.4) |  |
| **Agree** | 14 (45.2) | 37 (41.1) |  |
| **Strongly Agree** | 12 (38.7) | 29 (32.2) |  |
| **The department has done a good job providing training programs that promote understanding and mitigation of unconscious bias.** | | | |
| **Strongly Disagree** | 1 (3.2) | 4 (4.4) | 0.151 |
| **Disagree** | 3 (9.7) | 19 (21.1) |  |
| **Neither** | 7 (22.6) | 27 (30) |  |
| **Agree** | 13 (41.9) | 33 (36.7) |  |
| **Strongly Agree** | 7 (22.6) | 7 (7.8) |  |
| **Employees of different backgrounds are encouraged to apply for higher positions.** | | | |
| **Strongly Disagree** | 0 (0) | 3 (3.3) | 0.708 |
| **Disagree** | 1 (3.2) | 7 (7.8) |  |
| **Neither** | 11 (35.5) | 32 (35.6) |  |
| **Agree** | 11 (35.5) | 30 (33.3) |  |
| **Strongly Agree** | 8 (25.8) | 18 (20) |  |
| **There is a career development path for all employees in this department.** | | | |
| **Strongly Disagree** | 0 (0) | 7 (7.8) | 0.105 |
| **Disagree** | 3 (9.7) | 17 (18.9) |  |
| **Neither** | 10 (32.3) | 35 (38.9) |  |
| **Agree** | 11 (35.5) | 22 (24.4) |  |
| **Strongly Agree** | 7 (22.6) | 9 (10.0) |  |
| **I have received implicit bias or cultural sensitivity training before.** | | | |
| **Strongly Disagree** | 1 (3.2) | 3 (3.3) | 0.639 |
| **Disagree** | 1 (3.2) | 12 (13.3) |  |
| **Neither** | 2 (6.5) | 6 (6.7) |  |
| **Agree** | 20 (64.5) | 52 (57.8) |  |
| **Strongly Agree** | 7 (22.6) | 17 (18.9) |  |
| ***2023 (N=201)*** | | | |
| **n (%)** | **Men** | **Women** | **p-value** |
| **I can be my authentic self at work.** | | | |
| **Strongly Disagree** | 0 (0) | 5 (3.8) | 0.321 |
| **Disagree** | 1 (1.6) | 4 (3.1) |  |
| **Neither** | 5 (8.2) | 11 (8.5) |  |
| **Agree** | 29 (47.5) | 70 (53.8) |  |
| **Strongly Agree** | 26 (42.6) | 40 (30.8) |  |
| **My professional opinions and contributions are considered equally, regardless of my individual characteristics.** | | | |
| **Strongly Disagree** | 1 (1.6) | 5 (3.8) | 0.156 |
| **Disagree** | 5 (8.2) | 8 (6.2) |  |
| **Neither** | 4 (6.6) | 20 (15.4) |  |
| **Agree** | 25 (41) | 61 (46.9) |  |
| **Strongly Agree** | 26 (42.6) | 36 (27.7) |  |
| **I feel comfortable speaking out when I see examples of bias or non-inclusive behavior.** | | | |
| **Strongly Disagree** | 1 (1.6) | 4 (3.1) | 0.107 |
| **Disagree** | 8 (13.1) | 14 (10.8) |  |
| **Neither** | 5 (8.2) | 30 (23.1) |  |
| **Agree** | 29 (47.5) | 57 (43.8) |  |
| **Strongly Agree** | 18 (29.5) | 25 (19.2) |  |
| **I observe examples of bias or non-inclusive behavior on a regular basis in this department.** | | | |
| **Strongly Disagree** | 20 (32.8) | 31 (23.8) | 0.460 |
| **Disagree** | 21 (34.4) | 54 (41.5) |  |
| **Neither** | 9 (14.8) | 28 (21.5) |  |
| **Agree** | 7 (11.5) | 12 (9.2) |  |
| **Strongly Agree** | 4 (6.6) | 5 (3.8) |  |
| **The department provides an environment for the free and open expression of ideas, opinions and beliefs.** | | | |
| **Strongly Disagree** | 0 (0) | 2 (1.5) | 0.851 |
| **Disagree** | 4 (6.6) | 9 (6.9) |  |
| **Neither** | 13 (21.3) | 23 (17.7) |  |
| **Agree** | 29 (47.5) | 66 (50.8) |  |
| **Strongly Agree** | 15 (24.6) | 30 (23.1) |  |
| **I believe this department will take appropriate action in response to incidents of discrimination.** | | | |
| **Strongly Disagree** | 0 (0) | 3 (2.3) | 0.402 |
| **Disagree** | 2 (3.3) | 7 (5.4) |  |
| **Neither** | 12 (19.7) | 22 (16.9) |  |
| **Agree** | 20 (32.8) | 56 (43.1) |  |
| **Strongly Agree** | 27 (44.3) | 42 (32.3) |  |
| **My supervisor supports diversity by showing commitment to handling matters responsibly and appropriately.** | | | |
| **Strongly Disagree** | 0 (0) | 3 (2.3) | 0.627 |
| **Disagree** | 2 (3.3) | 5 (3.8) |  |
| **Neither** | 8 (13.1) | 18 (13.8) |  |
| **Agree** | 22 (36.1) | 54 (41.5) |  |
| **Strongly Agree** | 29 (47.5) | 50 (38.5) |  |
| **The department has done a good job providing training programs that promote understanding and mitigation of unconscious bias.** | | | |
| **Strongly Disagree** | 0 (0) | 0 (0) | 0.288 |
| **Disagree** | 0 (0) | 4 (3.1) |  |
| **Neither** | 10 (16.4) | 17 (13.1) |  |
| **Agree** | 26 (42.6) | 67 (51.5) |  |
| **Strongly Agree** | 25 (41) | 42 (32.3) |  |
| **Employees of different backgrounds are encouraged to apply for higher positions.** | | | |
| **Strongly Disagree** | 1 (1.6) | 5 (3.8) | 0.515 |
| **Disagree** | 3 (4.9) | 9 (6.9) |  |
| **Neither** | 15 (24.6) | 37 (28.5) |  |
| **Agree** | 21 (34.4) | 49 (37.7) |  |
| **Strongly Agree** | 21 (34.4) | 30 (23.1) |  |
| **There is a career development path for all employees in this department.** | | | |
| **Strongly Disagree** | 2 (3.3) | 7 (5.4) | 0.114 |
| **Disagree** | 6 (9.8) | 24 (18.5) |  |
| **Neither** | 14 (23) | 39 (30) |  |
| **Agree** | 21 (34.4) | 40 (30.8) |  |
| **Strongly Agree** | 18 (29.5) | 20 (15.4) |  |
| **I have received implicit bias or cultural sensitivity training before.** | | | |
| **Strongly Disagree** | 0 (0) | 1 (0.8) | **0.035** |
| **Disagree** | 1 (1.6) | 8 (6.2) |  |
| **Neither** | 5 (8.2) | 13 (10) |  |
| **Agree** | 25 (41) | 73 (56.2) |  |
| **Strongly Agree** | 30 (49.2) | 35 (26.9) |  |
| *^a^* Total number of departmental employees in 2021 and 2023 are 332 and 406, respectively. *^b^* Total number of missing sex responses was 14 (10.4%) in 2021 and 10 (5%) in 2023.  *^c^* Chi-square testing was used to assess differences between demogrphic categories. | | | |

| **Supplemental Table 3.** Department answers when surveys were given in 2021 and 2023 stratified by seniority, or years working within the department. | | | | | |
| --- | --- | --- | --- | --- | --- |
| ***2021 (N=135)* *^a,b^*** | | | | | |
| **Seniority n (%)** | **< 1 - 3 years** | **4 – 9 years** | **10+ years** | **p-value** *^c^* |  |
| **Strongly Disagree** | 0 (0) | 1 (1.8) | 0 (0) | 0.144 |  |
| **Disagree** | 0 (0) | 6 (10.9) | 1 (3.4) |  |  |
| **Neither** | 2 (4.9) | 10 (18.2) | 5 (17.2) |  |  |
| **Agree** | 24 (58.5) | 25 (45.5) | 15 (51.7) |  |  |
| **Strongly Agree** | 15 (36.6) | 13 (23.6) | 8 (27.6) |  |  |
| **My professional opinions and contributions are considered equally, regardless of my individual characteristics.** | | | | | |
| **Strongly Disagree** | 0 (0) | 4 (7.3) | 0 (0) | **0.007** |  |
| **Disagree** | 2 (4.9) | 8 (14.5) | 1 (3.4) |  |  |
| **Neither** | 2 (4.9) | 12 (21.8) | 2 (6.9) |  |  |
| **Agree** | 24 (58.5) | 22 (40) | 20 (16) |  |  |
| **Strongly Agree** | 13 (31.7) | 9 (16.4) | 6 (20.7) |  |  |
| **I feel comfortable speaking out when I see examples of bias or non-inclusive behavior.** | | | | | |
| **Strongly Disagree** | 0 (0) | 4 (7.3) | 0 (0) | **0.001** |  |
| **Disagree** | 3 (7.3) | 15 (27.3) | 5 (17.2) |  |  |
| **Neither** | 6 (14.6) | 15 (27.3) | 3 (10.3) |  |  |
| **Agree** | 25 (61) | 14 (25.5) | 11 (37.9) |  |  |
| **Strongly Agree** | 7 (17.1) | 7 (12.7) | 10 (34.5) |  |  |
| **I observe examples of bias or non-inclusive behavior on a regular basis in this department.** | | | | | |
| **Strongly Disagree** | 13 (31.7) | 8 (14.5) | 7 (24.1) | **0.011** |  |
| **Disagree** | 21 (51.2) | 16 (29.1) | 12 (41.4) |  |  |
| **Neither** | 3 (7.3) | 13 (23.6) | 4 (13.8) |  |  |
| **Agree** | 4 (9.8) | 12 (21.8) | 6 (20.7) |  |  |
| **Strongly Agree** | 0 (0) | 6 (10.9) | 0 (0) |  |  |
| **The department provides an environment for the free and open expression of ideas, opinions and beliefs.** | | | | | |
| **Strongly Disagree** | 0 (0) | 5 (9.1) | 1 (3.4) | **0.023** |  |
| **Disagree** | 1 (2.4) | 10 (18.2) | 1 (3.4) |  |  |
| **Neither** | 6 (14.6) | 12 (21.8) | 9 (31) |  |  |
| **Agree** | 23 (56.1) | 20 (36.4) | 12 (41.4) |  |  |
| **Strongly Agree** | 11 (26.8) | 8 (14.5) | 6 (20.7) |  |  |
| **I believe this department will take appropriate action in response to incidents of discrimination.** | | | | | |
| **Strongly Disagree** | 0 (0) | 4 (7.3) | 1 (3.4) | **0.003** |  |
| **Disagree** | 1 (2.4) | 7 (12.7) | 2 (6.9) |  |  |
| **Neither** | 2 (4.9) | 16 (29.1) | 8 (27.6) |  |  |
| **Agree** | 20 (48.8) | 19 (34.5) | 7 (24.1) |  |  |
| **Strongly Agree** | 18 (43.9) | 9 (16.4) | 11 (37.9) |  |  |
| **My supervisor supports diversity by showing commitment to handling matters responsibly and appropriately.** | | | | | |
| **Strongly Disagree** | 0 (0) | 2 (3.6) | 0 (0) | 0.100 |  |
| **Disagree** | 0 (0) | 10 (18.2) | 1 (3.4) |  |  |
| **Neither** | 3 (7.3) | 7 (12.7) | 9 (31) |  |  |
| **Agree** | 18 (43.9) | 23 (41.8) | 10 (34.5) |  |  |
| **Strongly Agree** | 20 (48.8) | 13 (23.6) | 9 (31) |  |  |
| **The department has done a good job providing training programs that promote understanding and mitigation of unconscious bias.** | | | | | |
| **Strongly Disagree** | 0 (0) | 6 (10.9) | 0 (0) | **0.012** |  |
| **Disagree** | 4 (9.8) | 9 (16.4) | 11 (37.9) |  |  |
| **Neither** | 12 (29.3) | 15 (27.3) | 7 (24.1) |  |  |
| **Agree** | 17 (41.5) | 21 (38.2) | 9 (31) |  |  |
| **Strongly Agree** | 8 (19.5) | 4 (7.3) | 2 (6.9) |  |  |
| **Employees of different backgrounds are encouraged to apply for higher positions.** | | | | | |
| **Strongly Disagree** | 1 (2.4) | 3 (5.5) | 0 (0) | 0.066 |  |
| **Disagree** | 0 (0) | 7 (12.7) | 0 (0) |  |  |
| **Neither** | 15 (36.6) | 21 (38.2) | 10 (34.5) |  |  |
| **Agree** | 14 (34.1) | 17 (30.9) | 10 (34.5) |  |  |
| **Strongly Agree** | 11 (26.8) | 7 (12.7) | 9 (31) |  |  |
| **There is a career development path for all employees in this department.** | | | | | |
| **Strongly Disagree** | 1 (2.4) | 9 (16.4) | 2 (6.9) | **0.044** |  |
| **Disagree** | 3 (7.3) | 13 (23.6) | 3 (10.3) |  |  |
| **Neither** | 15 (36.6) | 18 (32.7) | 14 (48.3) |  |  |
| **Agree** | 14 (34.1) | 11 (20) | 6 (20.7) |  |  |
| **Strongly Agree** | 8 (19.5) | 4 (7.3) | 4 (13.8) |  |  |
| **I have received implicit bias or cultural sensitivity training before.** | | | | | |
| **Strongly Disagree** | 2 (4.9) | 1 (1.8) | 1 (3.4) | 0.240 |  |
| **Disagree** | 1 (2.4) | 10 (18.2) | 2 (6.9) |  |  |
| **Neither** | 3 (7.3) | 4 (7.3) | 2 (6.9) |  |  |
| **Agree** | 24 (58.5) | 34 (61.8) | 17 (58.6) |  |  |
| **Strongly Agree** | 11 (26.8) | 6 (10.9) | 7 (24.1) |  |  |
| ***2023 (N=201)*** | | | | | |
| **Seniority n (%)** | **< 1 - 3 years** | **4 – 9 years** | **10+ years** | **p-value** | |
| **Strongly Disagree** | 1 (1.8) | 2 (2.2) | 2 (4.7) | 0.369 | |
| **Disagree** | 0 (0) | 4 (4.4) | 1 (2.3) |  |  |
| **Neither** | 7 (12.5) | 5 (5.5) | 4 (9.3) |  |  |
| **Agree** | 24 (42.9) | 51 (56) | 24 (55.8) |  |  |
| **Strongly Agree** | 24 (42.9) | 29 (31.9) | 12 (27.9) |  |  |
| **My professional opinions and contributions are considered equally, regardless of my individual characteristics.** | | | | | |
| **Strongly Disagree** | 1 (1.8) | 5 (5.5) | 0 (0) | 0.183 | |
| **Disagree** | 1 (1.8) | 8 (8.8) | 4 (9.3) |  |  |
| **Neither** | 4 (7.1) | 14 (15.4) | 5 (11.6) |  |  |
| **Agree** | 29 (51.8) | 35 (38.5) | 23 (53.5) |  |  |
| **Strongly Agree** | 21 (37.5) | 29 (31.9) | 11 (25.6) |  |  |
| **I feel comfortable speaking out when I see examples of bias or non-inclusive behavior.** | | | | | |
| **Strongly Disagree** | 0 (0) | 4 (4.4.) | 1 (2.3) | 0.509 | |
| **Disagree** | 7 (12.5) | 10 (11) | 5 (11.6) |  |  |
| **Neither** | 12 (21.4) | 17 (18.7) | 6 (14) |  |  |
| **Agree** | 22 (39.3) | 40 (44) | 25 (58.1) |  |  |
| **Strongly Agree** | 15 (26.8) | 20 (22) | 6 (14) |  |  |
| **I observe examples of bias or non-inclusive behavior on a regular basis in this department.** | | | | | |
| **Strongly Disagree** | 21 (37.5) | 22 (24.2) | 8 (18.6) | 0.053 | |
| **Disagree** | 22 (39.3) | 34 (37.4) | 18 (41.9) |  |  |
| **Neither** | 8 (14.3) | 20 (22) | 9 (20.9) |  |  |
| **Agree** | 4 (7.1) | 7 (7.7) | 8 (18.6) |  |  |
| **Strongly Agree** | 1 (1.8) | 8 (8.8) | 0 (0) |  |  |
| **The department provides an environment for the free and open expression of ideas, opinions and beliefs.** | | | | | |
| **Strongly Disagree** | 1 (1.8) | 1 (1.1) | 0 (0) | 0.438 | |
| **Disagree** | 1 (1.8) | 9 (9.9) | 3 (7.0) |  |  |
| **Neither** | 8 (14.3) | 17 (18.7) | 11 (25.6) |  |  |
| **Agree** | 29 (51.8) | 43 (47.3) | 22 (51.2) |  |  |
| **Strongly Agree** | 17 (30.4) | 21 (23.1) | 7 (16.3) |  |  |
| **I believe this department will take appropriate action in response to incidents of discrimination.** | | | | | |
| **Strongly Disagree** | 0 (0) | 3 (3.3) | 0 (0) | 0.418 | |
| **Disagree** | 1 (1.8) | 6 (6.6) | 2 (4.7) |  |  |
| **Neither** | 10 (17.9) | 13 (14.3) | 11 (25.6) |  |  |
| **Agree** | 22 (39.3) | 39 (42.9) | 15 (34.9) |  |  |
| **Strongly Agree** | 23 (41.1) | 30 (33) | 15 (34.9) |  |  |
| **My supervisor supports diversity by showing commitment to handling matters responsibly and appropriately.** | | | | | |
| **Strongly Disagree** | 1 (1.8) | 2 (2.2.) | 0 (0) | 0.839 | |
| **Disagree** | 1 (1.8) | 4 (4.4) | 2 (4.7) |  |  |
| **Neither** | 5 (8.9) | 14 (15.4) | 8 (18.6) |  |  |
| **Agree** | 24 (42.9) | 36 (39.6) | 15 (34.9) |  |  |
| **Strongly Agree** | 25 (44.6) | 35 (38.5) | 18 (41.9) |  |  |
| **The department has done a good job providing training programs that promote understanding and mitigation of unconscious bias.** | | | | | |
| **Strongly Disagree** | 0 (0) | 0 (0) | 0 (0) | 0.582 | |
| **Disagree** | 1 (1.8) | 1 (1.1) | 2 (4.7) |  |  |
| **Neither** | 6 (10.7) | 16 (17.6) | 6 (14) |  |  |
| **Agree** | 25 (44.6) | 45 (49.5) | 22 (51.2) |  |  |
| **Strongly Agree** | 24 (42.9) | 29 (31.9) | 13 (30.2) |  |  |
| **Employees of different backgrounds are encouraged to apply for higher positions.** | | | | | |
| **Strongly Disagree** | 1 (1.8) | 5 (5.5.) | 0 (0) | 0.655 | |
| **Disagree** | 3 (5.4) | 5 (5.5) | 4 (9.3) |  |  |
| **Neither** | 16 (28.6) | 27 (29.7) | 9 (20.9) |  |  |
| **Agree** | 21 (37.5) | 33 (36.3) | 16 (37.2) |  |  |
| **Strongly Agree** | 15 (26.8) | 21 (23.1) | 14 (32.6) |  |  |
| **There is a career development path for all employees in this department.** | | | | | |
| **Strongly Disagree** | 4 (7.1) | 5 (5.5) | 0 (0) | 0.070 | |
| **Disagree** | 3 (5.4) | 17 (18.7) | 10 (23.3) |  |  |
| **Neither** | 13 (23.2) | 25 (27.5) | 14 (32.6) |  |  |
| **Agree** | 21 (37.5) | 25 (27.5) | 15 (34.9) |  |  |
| **Strongly Agree** | 15 (26.8) | 19 (20.9) | 4 (9.3) |  |  |
| **I have received implicit bias or cultural sensitivity training before.** | | | | | |
| **Strongly Disagree** | 1 (1.8) | 0 (0) | 0 (0) | 0.268 | |
| **Disagree** | 4 (7.1) | 2 (2.2) | 2 (4.7) |  |  |
| **Neither** | 6 (10.7) | 7 (7.7) | 5 (11.6) |  |  |
| **Agree** | 22 (39.3) | 51 (56) | 26 (60.5) |  |  |
| **Strongly Agree** | 23 (41.1) | 31 (34.1) | 10 (23.3) |  |  |
| *^a^* Total number of departmental employees in 2020 and 2023 are 332 and 406, respectively. *^b^* Total number of missing seniority responses was 10 (7.4%) in 2021 and 11 (5.5%) in 2023.  *^c^* Chi-square testing was used to assess differences between demographic categories. | | | | | |

| **Supplemental Table 4.** Department answers when surveys were given in 2021 and 2023 stratified by location. | | | | |
| --- | --- | --- | --- | --- |
| ***2021 (N=135)* *^a,b^*** | | | | |
| **n (%)** | **Main Campus** | **Community Sites** | **p-value** *^c^* |  |
| **I can be my authentic self at work.** | | | | |
| **Strongly Disagree** | 1 (1.2) | 0 (0) | 0.351 |  |
| **Disagree** | 5 (5.8) | 2 (4.1) |  |  |
| **Neither** | 13 (15.1) | 9 (18.4) |  |  |
| **Agree** | 39 (45.3) | 29 (59.2) |  |  |
| **Strongly Agree** | 28 (32.6) | 9 (18.4) |  |  |
| **My professional opinions and contributions are considered equally, regardless of my individual characteristics.** | | | | |
| **Strongly Disagree** | 1 (1.2) | 3 (6.1) | 0.382 |  |
| **Disagree** | 7 (8.1) | 6 (12.2) |  |  |
| **Neither** | 11 (12.8) | 7 (14.3) |  |  |
| **Agree** | 46 (53.5) | 25 (51.0) |  |  |
| **Strongly Agree** | 21 (24.4) | 8 (16.3) |  |  |
| **I feel comfortable speaking out when I see examples of bias or non-inclusive behavior.** | | | | |
| **Strongly Disagree** | 1 (1.2) | 3 (6.1) | 0.286 |  |
| **Disagree** | 13 (15.1) | 12 (24.5) |  |  |
| **Neither** | 21 (24.4) | 10 (20.4) |  |  |
| **Agree** | 34 (39.5) | 17 (34.7) |  |  |
| **Strongly Agree** | 17 (19.8) | 7 (14.3) |  |  |
| **I observe examples of bias or non-inclusive behavior on a regular basis in this department.** | | | | |
| **Strongly Disagree** | 16 (18.6) | 12 (24.5) | **0.002** |  |
| **Disagree** | 43 (50) | 9 (18.4) |  |  |
| **Neither** | 10 (11.6) | 13 (26.5) |  |  |
| **Agree** | 15 (17.4) | 10 (20.4) |  |  |
| **Strongly Agree** | 2 (2.3) | 5 (10.2) |  |  |
| **The department provides an environment for the free and open expression of ideas, opinions and beliefs.** | | | | |
| **Strongly Disagree** | 2 (2.3) | 5 (10.2) | 0.084 |  |
| **Disagree** | 6 (7.0) | 7 (14.3) |  |  |
| **Neither** | 22 (25.6) | 9 (18.4) |  |  |
| **Agree** | 36 (41.9) | 22 (44.9) |  |  |
| **Strongly Agree** | 20 (23.3) | 6 (12.2) |  |  |
| **I believe this department will take appropriate action in response to incidents of discrimination.** | | | | |
| **Strongly Disagree** | 3 (3.5) | 2 (4.1) | 0.168 |  |
| **Disagree** | 8 (9.3) | 4 (8.2) |  |  |
| **Neither** | 14 (16.3) | 16 (32.7) |  |  |
| **Agree** | 32 (37.2) | 18 (36.7) |  |  |
| **Strongly Agree** | 29 (33.7) | 9 (18.4) |  |  |
| **My supervisor supports diversity by showing commitment to handling matters responsibly and appropriately.** | | | | |
| **Strongly Disagree** | 0 (0) | 2 (4.1) | 0.153 |  |
| **Disagree** | 7 (8.1) | 4 (8.2) |  |  |
| **Neither** | 13 (15.1) | 9 (18.4) |  |  |
| **Agree** | 33 (38.4) | 23 (46.9) |  |  |
| **Strongly Agree** | 33 (38.4) | 11 (22.4) |  |  |
| **The department has done a good job providing training programs that promote understanding and mitigation of unconscious bias.** | | | | |
| **Strongly Disagree** | 4 (4.7) | 2 (4.1) | **0.023** |  |
| **Disagree** | 10 (11.6) | 15 (30.6) |  |  |
| **Neither** | 23 (26.7) | 16 (32.7) |  |  |
| **Agree** | 36 (41.9) | 14 (28.6) |  |  |
| **Strongly Agree** | 13 (15.1) | 2 (4.1) |  |  |
| **Employees of different backgrounds are encouraged to apply for higher positions.** | | | | |
| **Strongly Disagree** | 2 (2.3) | 3 (6.1) | 0.613 |  |
| **Disagree** | 6 (7.0) | 2 (4.1) |  |  |
| **Neither** | 30 (34.9) | 20 (40.8) |  |  |
| **Agree** | 28 (32.6) | 16 (32.7) |  |  |
| **Strongly Agree** | 20 (23.3) | 8 (16.3) |  |  |
| **There is a career development path for all employees in this department.** | | | | |
| **Strongly Disagree** | 5 (5.8) | 7 (14.3) | 0.125 |  |
| **Disagree** | 11 (12.8) | 10 (20.4) |  |  |
| **Neither** | 31 (36.0) | 19 (38.8) |  |  |
| **Agree** | 25 (29.1) | 10 (20.4) |  |  |
| **Strongly Agree** | 14 (16.3) | 3 (6.1) |  |  |
| **I have received implicit bias or cultural sensitivity training before.** | | | | |
| **Strongly Disagree** | 1 (1.2) | 3 (6.1) | 0.358 |  |
| **Disagree** | 9 (10.5) | 5 (10.2) |  |  |
| **Neither** | 6 (7.0) | 4 (8.2) |  |  |
| **Agree** | 51 (59.3) | 31 (63.3) |  |  |
| **Strongly Agree** | 19 (22.1) | 6 (12.2) |  |  |
| ***2023 (N=201)*** | | | | |
| **n (%)** | **Main Campus** | **Community Sites** | **p-value** |  |
| **I can be my authentic self at work.** | | | | |
| **Strongly Disagree** | 5 (3.6) | 0 (0) | 0.139 |  |
| **Disagree** | 4 (2.9) | 1 (2.0) |  |  |
| **Neither** | 16 (11.7) | 1 (2.0) |  |  |
| **Agree** | 67 (48.9) | 31 (60.8) |  |  |
| **Strongly Agree** | 45 (32.8) | 18 (35.3) |  |  |
| **My professional opinions and contributions are considered equally, regardless of my individual characteristics.** | | | | |
| **Strongly Disagree** | 5 (3.6) | 1 (2.0) | 0.311 |  |
| **Disagree** | 10 (7.3) | 2 (3.9) |  |  |
| **Neither** | 21 (15.3) | 3 (5.9) |  |  |
| **Agree** | 57 (41.6) | 27 (52.9) |  |  |
| **Strongly Agree** | 44 (32.1) | 18 (35.3) |  |  |
| **I feel comfortable speaking out when I see examples of bias or non-inclusive behavior.** | | | | |
| **Strongly Disagree** | 4 (2.9) | 1 (2.0) | 0.883 |  |
| **Disagree** | 17 (12.4) | 4 (7.8) |  |  |
| **Neither** | 25 (18.2) | 10 (19.6) |  |  |
| **Agree** | 62 (45.3) | 23 (45.1) |  |  |
| **Strongly Agree** | 29 (21.2) | 13 (25.5) |  |  |
| **I observe examples of bias or non-inclusive behavior on a regular basis in this department.** | | | | |
| **Strongly Disagree** | 36 (19.1) | 15 (29.4) | 0.744 |  |
| **Disagree** | 55 (40.1) | 18 (35.3) |  |  |
| **Neither** | 26 (19.0) | 12 (23.5) |  |  |
| **Agree** | 12 (8.8) | 5 (9.8) |  |  |
| **Strongly Agree** | 8 (5.8) | 1 (2.0) |  |  |
| **The department provides an environment for the free and open expression of ideas, opinions and beliefs.** | | | | |
| **Strongly Disagree** | 1 (0.7) | 1 (2.0) | 0.276 |  |
| **Disagree** | 11 (8.0) | 2 (3.9) |  |  |
| **Neither** | 29 (21.2) | 5 (9.8) |  |  |
| **Agree** | 65 (47.4) | 29 (56.9) |  |  |
| **Strongly Agree** | 31 (22.6) | 14 (27.5) |  |  |
| **I believe this department will take appropriate action in response to incidents of discrimination.** | | | | |
| **Strongly Disagree** | 2 (1.5) | 1 (2.0) | 0.718 |  |
| **Disagree** | 7 (5.1) | 2 (3.9) |  |  |
| **Neither** | 27 (19.7) | 6 (11.8) |  |  |
| **Agree** | 51 (37.2) | 23 (45.1) |  |  |
| **Strongly Agree** | 50 (36.5) | 17 (37.3) |  |  |
| **My supervisor supports diversity by showing commitment to handling matters responsibly and appropriately.** | | | | |
| **Strongly Disagree** | 3 (2.2) | 0 (0) | 0.584 |  |
| **Disagree** | 4 (2.9) | 2 (3.9) |  |  |
| **Neither** | 20 (14.6) | 4 (7.8) |  |  |
| **Agree** | 54 (39.4) | 22 (43.1) |  |  |
| **Strongly Agree** | 56 (40.9) | 23 (45.1) |  |  |
| **The department has done a good job providing training programs that promote understanding and mitigation of unconscious bias.** | | | | |
| **Strongly Disagree** | 0 (0) | 0 (0) | 0.576 |  |
| **Disagree** | 3 (2.2) | 0 (0) |  |  |
| **Neither** | 20 (14.6) | 5 (9.8) |  |  |
| **Agree** | 66 (48.2) | 27 (52.9) |  |  |
| **Strongly Agree** | 48 (35.0) | 19 (37.3) |  |  |
| **Employees of different backgrounds are encouraged to apply for higher positions.** | | | | |
| **Strongly Disagree** | 6 (4.4) | 0 (0) | 0.056 |  |
| **Disagree** | 12 (8.8) | 0 (0) |  |  |
| **Neither** | 40 (29.2) | 12 (23.5) |  |  |
| **Agree** | 45 (32.8) | 22 (43.1) |  |  |
| **Strongly Agree** | 34 (24.8) | 17 (33.3) |  |  |
| **There is a career development path for all employees in this department.** | | | | |
| **Strongly Disagree** | 8 (5.8) | 0 (0) | 0.184 |  |
| **Disagree** | 19 (13.9) | 11 (21.6) |  |  |
| **Neither** | 36 (26.3) | 16 (31.4) |  |  |
| **Agree** | 43 (31.4) | 17 (33.3) |  |  |
| **Strongly Agree** | 31 (22.6) | 7 (13.7) |  |  |
| **I have received implicit bias or cultural sensitivity training before.** | | | | |
| **Strongly Disagree** | 0 (0) | 1 (2.0) | 0.104 |  |
| **Disagree** | 6 (4.4) | 3 (5.9) |  |  |
| **Neither** | 16 (11.7) | 2 (3.9) |  |  |
| **Agree** | 64 (46.7) | 31 (60.8) |  |  |
| **Strongly Agree** | 51 (37.2) | 14 (27.5) |  |  |

| *^a^* Total number of departmental employees in 2021 and 2023 are 332 and 406, respectively. *^b^* Total number of missing location responses was none in 2021 and 13 (6.5%) in 2023.  *^c^* Chi-square testing was used to assess differences between demographic categories. |
| --- |
